# Bilateral extensor pollicis et indicis accessorius: clinical and anatomical perspectives

**DOI:** 10.1007/s00276-026-03858-2

**Published:** 2026-04-07

**Authors:** Oluwabusayo A. Oni, Wren Adams, Preethi Prem, Ramya M. Ramakrishnan, Antara Sira, Emily A. Vachon, Mark T. Shima, Wessam Ibrahim

**Affiliations:** Carle Illinois College of Medicine, University of Illinois Urbana-Champaign, 506 South Mathews Ave., Urbana, IL 61801 USA

**Keywords:** Extensor pollicis longus, Extensor indicis, Extensor pollicis et indicis accessorius, Anatomical variation, Accessory muscle, Body donor dissection

## Abstract

**Purpose:**

The extensor muscles of the hand play a crucial role in thumb and finger movement. Thumb extension is primarily mediated by the extensor pollicis longus muscle and extensor pollicis brevis muscle, and extension of the index finger is facilitated by the extensor indicis muscle and extensor digitorum muscle. This report describes a rare anatomical variation, the extensor pollicis et indicis accessorius, in which a single tendon bifurcated to insert on both the index finger and thumb.

**Methods:**

This variation was identified bilaterally during routine dissection of a 92-year-old female donor. The donor had been embalmed with a formaldehyde-based fixation method.

**Results:**

On each side, an accessory extensor pollicis et indicis accessorius muscle was present in the deep posterior compartment of the forearm, giving rise to a single tendon that bifurcated distally to insert on both the dorsal aspect of the thumb and the dorsal expansion of the index finger. Measurements of the extensor pollicis et indicis accessorius muscle were not significantly different. Several case studies have documented the existence of such anomalies, but bilateral descriptions are rare.

**Conclusion:**

Historical and contemporary sources differ in defining extensor pollicis et indicis accessorius. Based on a comprehensive literature review and the present bilateral anatomical finding, this report recommends using the term extensor pollicis et indicis accessorius muscle for this configuration and proposes revising existing classification systems to include accessory extensor muscles originating from the extensor pollicis longus muscle, the extensor indicis muscle, or the ulna, rather than limiting them to extensor pollicis longus muscle anomalies.

**Supplementary Information:**

The online version contains supplementary material available at 10.1007/s00276-026-03858-2.

## Introduction

The extensor tendons of the hand are essential for coordinated thumb and finger movements. Thumb extension is produced by the combined action of the extensor pollicis longus muscle, which extends the interphalangeal and metacarpophalangeal joints of the thumb, and the extensor pollicis brevis muscle, which acts mainly at the metacarpophalangeal joint, often with additional contribution from the abductor pollicis longus muscle during radial deviation and composite thumb movements. Extension of the index finger is achieved through the extensor digitorum muscle and the extensor indicis muscle, which together provide independent extension and fine motor control at the metacarpophalangeal and interphalangeal joints of the index finger. These muscles act in concert rather than in isolation, and their relative mechanical contribution depends on joint position and specific task demands. Although these tendons are consistently present in humans, anatomical variations are not uncommon and can have significant clinical implications, particularly in surgical planning and tendon reconstruction. Terminology surrounding these variants has been inconsistent, with different authors using overlapping names for similar anatomical structures.

One notable variation is the presence of accessory muscles such as the extensor pollicis et indicis accessorius (EPIA), which provides a single tendon serving both the thumb and index finger. Although several reports have described such anomalies [10], bilateral occurrences are exceedingly rare and often complicated by inconsistent terminology in the literature. In particular, many reports do not distinguish between the extensor pollicis et indicis communis (EPIC) and the EPIA, despite differences in their reported origins, insertion patterns, and relationship to the EPL and EIM. Existing classification systems typically group these anomalies under variants of the EPL, yet historical descriptions suggest that these accessory muscles may originate from multiple sources, including the EPL, EIM, or ulna. Clarifying this distinction is necessary to avoid mislabeling and improve the comparability of future case reports.

In this report, we describe a bilateral EPIA identified during routine body donor dissection and review the historical and contemporary literature to clarify nomenclature and propose an amendment to the current classification system. Recognizing these variations is critical for accurate anatomical understanding and optimizing surgical outcomes.

## Methods

**Fig. 1:**
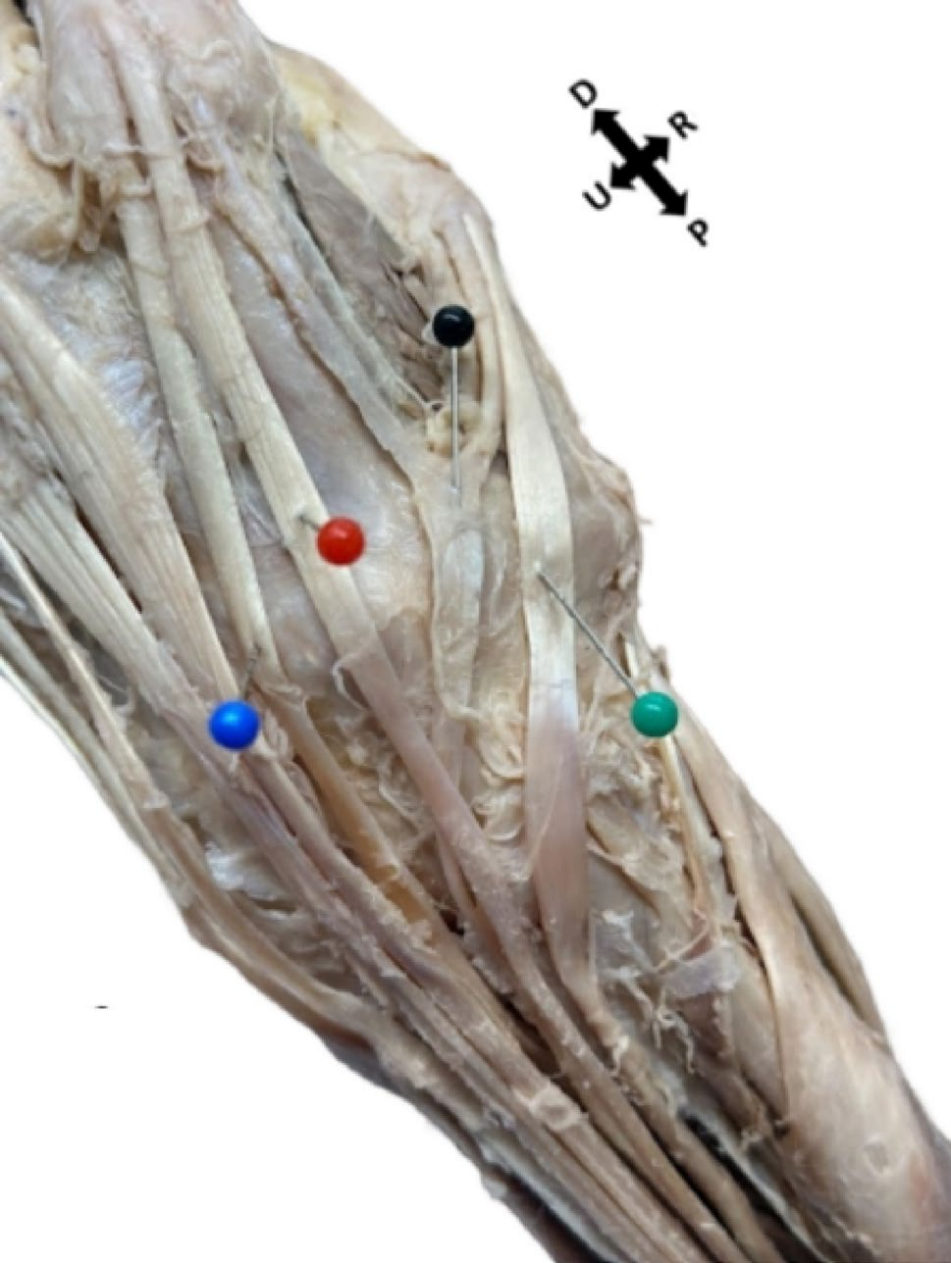
Extensor pollicis et indicis accessorius of the left hand. Black pin: Bilateral extensor pollicis et indicis accessorius Red pin: Extensor digitorum. Blue pin: Extensor indicis muscle. Green pin: Extensor pollicis longus. Directional arrows indicate proximal (P) and distal (D) radial (R) and ulnar (U) orientations

**Fig. 2:**
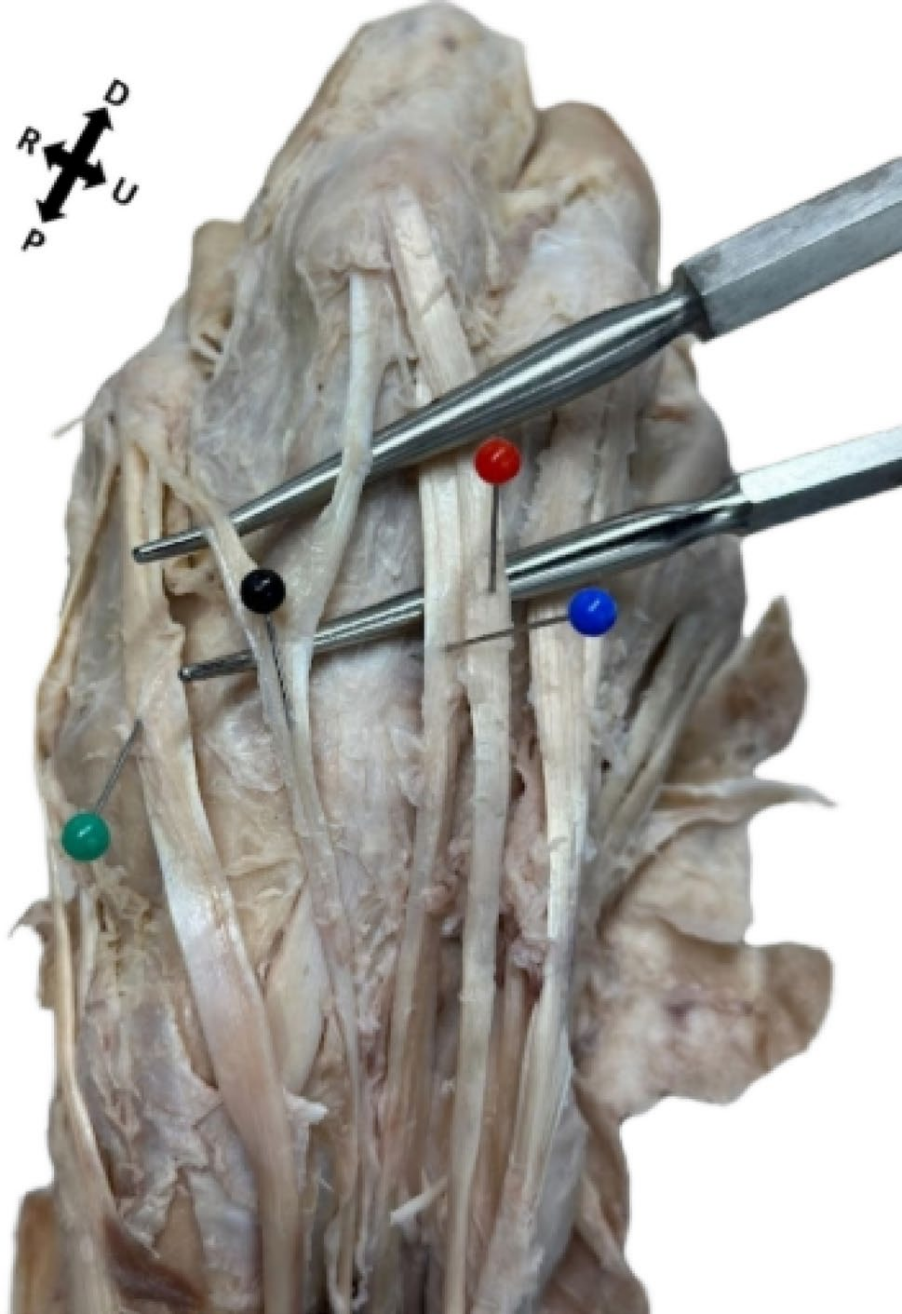
Extensor pollicis et indicis accessorius of the right hand. Black pin: Bilateral extensor pollicis et indicis accessorius. Red pin: Extensor digitorum. Blue pin: Extensor indicis muscle. Green pin: Extensor pollicis longus. Directional arrows indicate proximal (P) and distal (D) radial (R) and ulnar (U) orientations

**Fig. 3:**
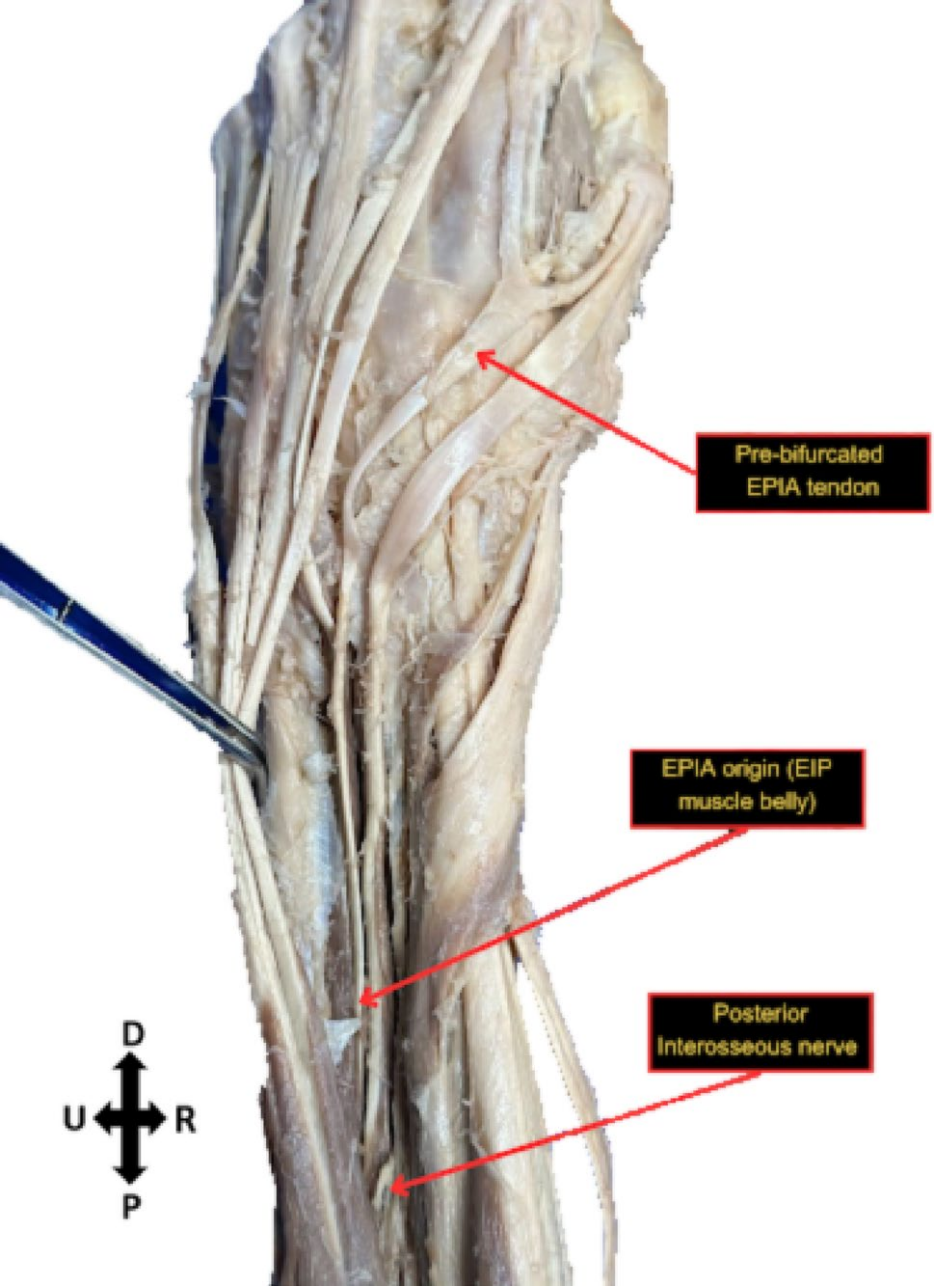
Origin and innervation of left extensor pollicis et indicis accessorius. Directional arrows indicate proximal (P) and distal (D) radial (R) and ulnar (U) orientations

**Fig. 4:**
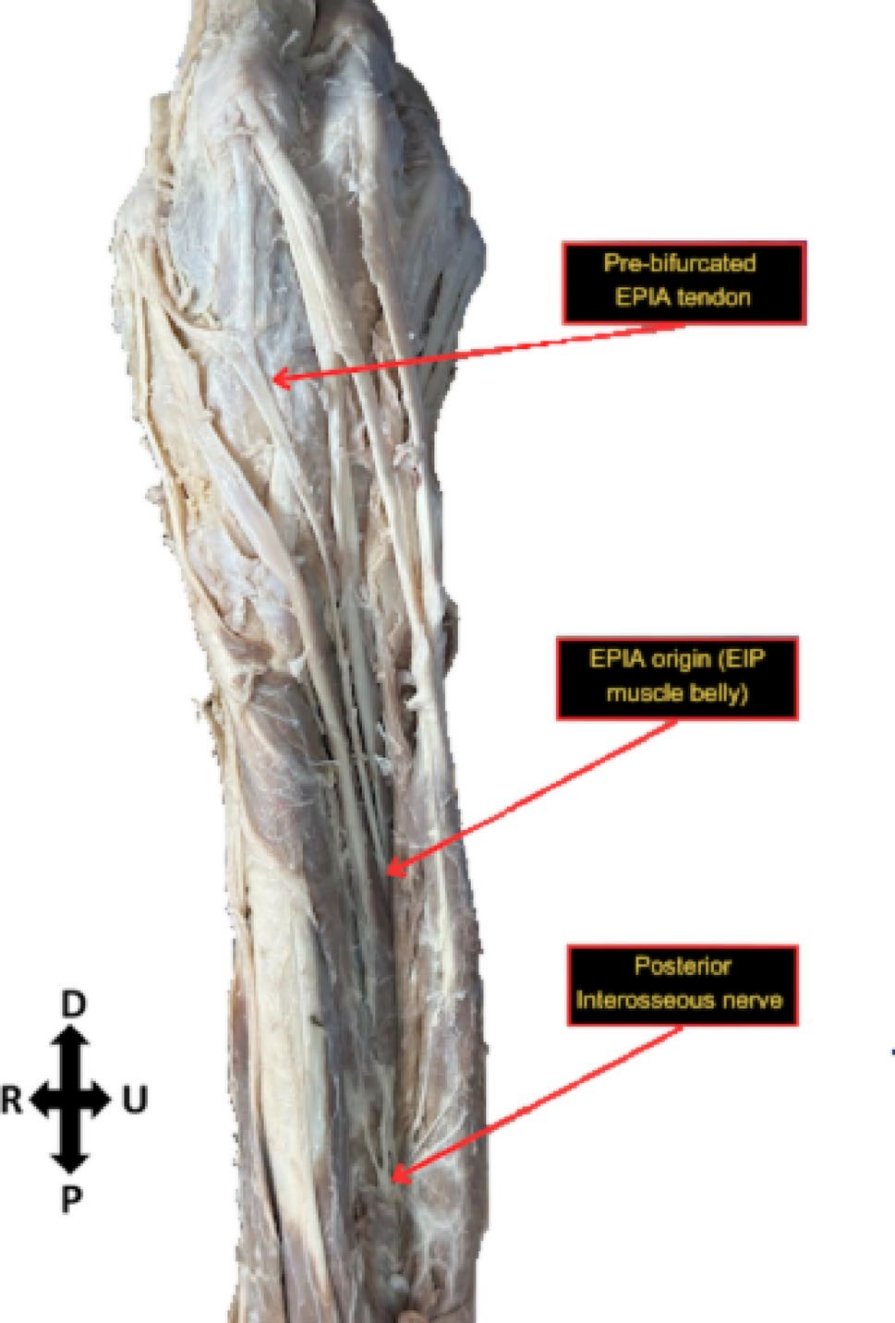
Origin and innervation of right extensor pollicis et indicis accessorius. Directional arrows indicate proximal (P) and distal (D) radial (R) and ulnar (U) orientations

Dissection was performed on a 92-year-old female body donor who died due to diastolic heart failure. The body donor was preserved through a standard formaldehyde-based arterial embalming with cavity fixation. Additionally notable anomalies of the body donor, not pictured, include tortuous common carotid arteries, a left-dominant posterior interventricular artery, and a bony mass on the left 12th rib. The extensor compartments were approached during standard dorsal wrist and hand dissection to expose the extensor retinaculum and the underlying tendons. The accessory tendons were recognized after the retinacular segments had already been disrupted. Consequently, the precise dorsal compartmental course could not be confirmed in situ. The posterior interosseous artery (PIA) typically supplies the deep extensor musculature, we were unable to document the vascular pedicle to the accessory muscle in this case, as its muscular branch from PIA had been partially disrupted. This anatomical variation was identified during the required first‑year medical gross anatomy labs at the Carle Illinois College of Medicine. The dissection was performed as part of routine curricular activities by a team of six first‑year medical students under faculty supervision (Figs. 1, 2, 3, 4).

## Case report

**Fig. 5:**
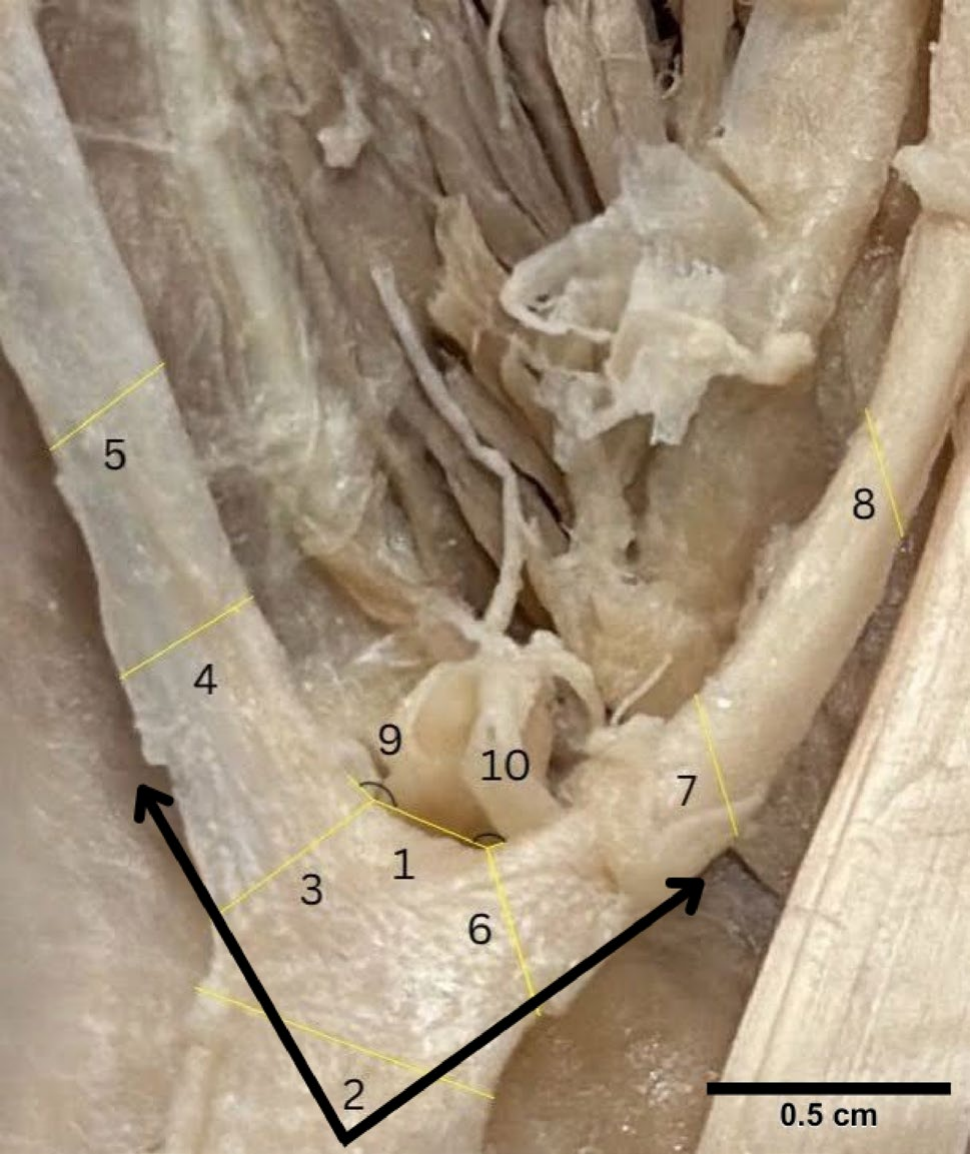
Measurements of bilateral extensor pollicis et indicis accessorius on the left hand. Numbers correspond to measurements (supplemental) Black arrows point towards tendon insertion sites

**Fig. 6:**
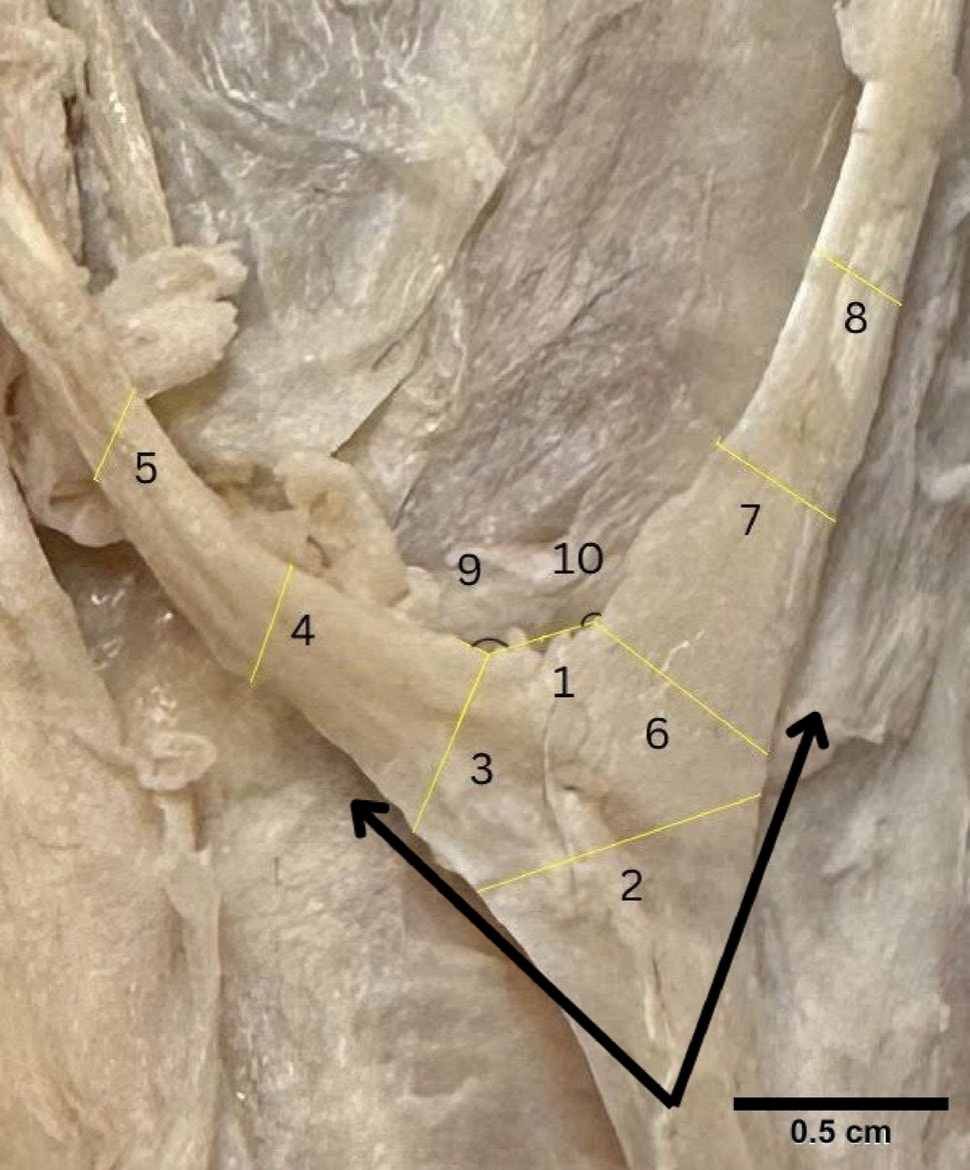
Measurements of bilateral extensor pollicis et indicis accessorius on the right hand. Numbers correspond to measurements (supplemental) Black arrows point towards tendon insertion sites

Upon dissection, an anomaly was noted bilaterally on the extensor side of both of the body donor’s hands. The body donor had bilateral accessory extensor tendons originating from the belly of the extensor indicis muscle that bifurcated into a medial index tendon and a lateral pollex tendon. Bilaterally, they are both innervated by the posterior interosseous nerve. The bilateral extensor pollicis et indicis accessorius was measured using ImageJ (see Supplementary Tables 1 and 2). Measurements marked on Figs. 5 and 6 were standardized with equal spacing and parallel orientation between corresponding measurement points on each side. Comparing the left and right hand respectively, the bases of the bifurcation were similar, 0.67 cm and 0.698 cm. A paired t-test comparing the bilateral extensor pollicis et indicis accessorius measurements demonstrated no statistically significant difference (*p* = 0.7466).

## Discussion

The extensor pollicis longus (EPL) is a deep muscle of the posterior forearm responsible for extending the thumb. The muscle originates from the posterior surface of the middle third of the ulna and the interosseous membrane of the forearm and inserts at the base of the distal phalanx of the thumb. Functionally, the EPL extends the thumb at the interphalangeal joint and at both the metacarpophalangeal and carpometacarpal joints. Additionally, the muscle contributes to thumb abduction at the wrist, enabling coordinated thumb movements essential for grip and manipulation [15]. The extensor indicis is another deep muscle located in the posterior aspect of the forearm. It originates from the posterior surface of the distal portion of the ulna and interosseous membrane of the forearm and inserts into the extensor expansion of the index finger. The primary function of the extensor indicis is to extend the index finger at the metacarpophalangeal joint, while also assisting in extension at the proximal and distal interphalangeal joints. This muscle provides an independent extension of the index finger, enabling more precise finger movements, and also plays a minor role in wrist extension [15]. In this case, routine dissection of the body donor revealed an extensor indicis muscle with a tendon that bifurcated into medial index and lateral pollex tendons. Supervised dissection of the donor’s second forearm by medical students revealed bilateral anomalies.

The extensor indicis tendon exhibits considerable variation in the human population. A recent systematic review and meta-analysis by Yammine et al. analyzed 29 body donor studies and classified the extensor indicis and its variants [14]. In this review, the extensor pollicis et indicis is referred to as the EPI. Nineteen of the 29 studies reported instances of an EPI, with a pooled prevalence estimate (PPE) of 0.75%. The prevalence of an EPI also appears to vary by geographic region, with European populations showing higher rates compared to Japanese and Indian populations.

The base width of the extensor pollicis et indicis accessorius measured 6.7 mm in the left hand and 6.98 mm in the right. In another anatomical case, these values exceed reported extensor indicis muscle widths, which measure approximately 3.4–4.4 mm for the combined radial and ulnar slip [1]. Although EPIA measurements were taken at the proximal base rather than along the free tendon, the larger size compared with both EIM slips suggests that this accessory structure may play a meaningful role in extensor function. Because the measurements were obtained from different anatomical locations, direct comparisons are limited and should be interpreted as estimates of relative size.

### Evolution and embryological development

The development of accessory muscles and tendons in the hand is thought to be influenced by embryological factors, although the exact mechanisms remain unclear. Some researchers suggest that these variations may be due to incomplete regression or excessive differentiation of the mesenchymal tissue during embryonic development [10]. Genetic factors and evolutionary adaptations have also been proposed as potential contributors. While these anomalies are typically asymptomatic, they may become clinically significant in cases of trauma, surgery, or degenerative conditions affecting the hand. Further research is needed to fully understand the factors contributing to the formation of these accessory structures and their potential functional implications.

The EIM and EPL are thought to have developed phylogenetically from the EPIA., mainly due to the fact that an EPIA has never been found in humans without a coexisting EIM and EPL. Gaulke also makes the argument that the development of independent thumb and index finger movement confers an evolutionary advantage [3]. The EIM, EPL, and extensor variants, such as the EPIC, derive from a structure deemed the extensor digitorum profundus mass. The extensor digitorum profundus mass is not a single muscle, but instead is a group of muscles found in nearly all mammals [8]. It varies significantly between species, but in primates, it almost always divides into two groups: an EPL and an extensor proprius. In humans, the extensor proprius is the EIM, but in other primates, the extensor proprius may extend to virtually any combination of digits I-V [8]. This high level of phylogenetic variability makes the extensor muscles prone to forming atypical variants.

Embryologically, all of the aforementioned extensor muscles derive from the extensor myotome. During muscle development, groups of myoblasts move from each myotome to produce specific muscles. Anomalies in this process can produce absent, accessory, or anomalous muscles. Additionally, errors in mesenchymal signaling may have similar effects [7].

### Clinical and functional implications

The EIM is often used as a donor tendon in hand surgeries because it has its own muscle belly. Additionally, the extensor digitorum muscle can compensate for the loss of EIM function. The accessory tendon in our body donor appears to arise from the same muscle belly as the EIM, which may make isolating and relocating the EIM tendon in surgery more challenging. On the other hand, the EPIA could potentially be utilized as another donor tendon if the EIM is damaged or insufficient [7].

In many cases, patients are likely unaware they possess accessory hand muscles. The extensor pollicis et indicis accessorius may confer above-average strength in the thumb and index fingers, but this may go unnoticed by the patient. Although we cannot determine our patient’s functional capabilities, the presence of an EPIA may compromise the independent movement of the thumb and index finger [7].

This accessory structure may also have made the patient more susceptible to certain pathologies, such as EIM syndrome, whole extensor compartment syndrome, and posterior interosseous nerve (PIN) entrapment. Ritter and Inglis define EIM syndrome as tenosynovitis of the EIM [6]. Ritter and Inglis note that the fourth extensor compartment is very small, especially when the fingers are flexed. Therefore, any increase in the size of the contents of the compartment may result in EIM syndrome. It follows that an EPIA could increase the risk of this pathology. Similarly, in cases of major wrist trauma, an accessory extensor muscle or tendon may increase the risk of whole extensor compartment syndrome [7]. Additionally, the PIN runs through the fourth extensor compartment. When an EPIA is present, the PIN may even course through the EPIA [7]. These scenarios make the PIN more prone to painful entrapment, a condition deemed posterior interosseous nerve syndrome.

### Inconsistencies in naming conventions and classification

We report a case of bilateral accessory extensor tendons of the hand found in a 92-year-old female body donor. These tendons have origins from the extensor indicis muscle, follow along the course of the EIM to the wrist, then split to have insertions at the extensor hoods of the index finger and thumb along with the EIM and EPL, respectively.

However, the precise naming of these accessory tendons is difficult to determine from the existing literature. Türker et al. propose a classification system for anomalies of the extensor pollicis longus [11]. It is worth noting that the specific cases presented by Türker et al. are from surgical procedures—therefore, the exact origins and insertions of the anomalies could not be directly visualized. The other variants identified by Türker et al. are derived from a number of sources in the literature, with many of these sources using conflicting criteria to name the anomalies presented. In this respect, Türker et al. correctly identified a need for a unified classification system for extensor tendon anomalies in order to resolve these conflicts. However, in our attempts to determine the proper name of the anomalies found in our body donor, we found that Türker et al. appear to have had a differing interpretation of the literature describing the extensor pollicis et indicis accessorius (EPIA) and that, in fact, there is no substantive difference between the EPIA and the extensor pollicis et indicis communis (EPIC). Furthermore, we found little reason to consider these accessory tendons to be variants of the EPL specifically: other classification systems consider these anomalies to be variants of the extensor indicis muscle, while still others consider them to be distinct muscles with distinct origins lying between the EIM and EPL.

The classification system described by Türker et al., as written, would have us name our body donor’s accessory tendons as EPICs. They describe the EPIC as “a supernumerary tendon which exited from the fourth dorsal compartment” and “initially followed a parallel course to the EPL towards the first web space… [then] split into two branches, with one attaching to the radial side of the extensor hood of the index finger and the other to the distal phalanx of the thumb” [11]. The same could largely be said of the accessory tendons in our body donor. Though we are unable to confirm which dorsal compartment our accessory tendons run through—the extensor retinacula were disrupted before the presence of these tendons was discovered—our tendons otherwise follow the same course as described by Türker et al.

The problem arises when we look closer at Türker et al. 's description of the “extensor pollicis et indicis accessories [sic]”. They state that the “accessories” tendon is an “interconnection between the EPL and [EIM],” citing Komiyama et al. [4]. Their interpretation of Komiyama seems to be that the “accessories” tendon has an origin from the EIM at the level of the wrist and only slightly distally inserts onto the EPL [11].

In fact, Komiyama et al. are referring to the extensor pollicis et indicis *accessorius*, and state that it is a supernumerary radial tendon of the extensor indicis muscle that “bifurcated at the middle level of the metacarpus,” with slips having insertions at “the radial side of the dorsum of the index finger” and “the tendon of the extensor pollicis longus” [11]. Unlike Türker et al., the diagram of Komiyama et al. depicts the EPIA having an origin from the muscle belly of the EIM; the tendon portion of the EIM remains independent of the EPIA until they both reach the extensor hood of the index finger. Furthermore, the EPIA has a separate tendon from both the EPL and EIM at the wrist level, which is not reflected in Türker et al.’s interpretation.

Komiyama’s reference for their description of the EPIA is Yoshida [15]. Whereas both Türker and Komiyama describe the EPIA as a tendon, Yoshida considers the EPIA to be a supernumerary muscle that lies between the EPL and EIM. The EPIA, as defined by Yoshida, has a single tendon that “bifurcates in the hand to supply the thumb and the index finger,” and that “the radial portion of the tendon is usually united with the extensor pollicis longus tendon at the distal part, and the ulnar portion is attached to the radial dorsum of the index finger.” Notably, Yoshida states that although the muscle belly of the EPIA is most often separated, variants where the muscle belly was fused with that of the EIM or EPL were still considered to be an EPIA [15]. Structurally, Komiyama’s diagram matches that of Yoshida, but Komiyama et al. portray the bifurcation to be much more horizontal. That, combined with Komiyama’s use of the word “slip,” seems to be the origin of Türker et al. 's interpretation: the EPIA, by its original meaning, matches Türker’s definition of the EPIC.

Türker cites Chiu when naming the EPIC, although Chiu primarily calls the structure simply an “extensor pollicis et indicis” [2]. It is only referred to as the extensor pollicis et indicis communis once, in a parenthetical as part of a figure caption. Chiu cites Wood (1867) and Macalister (1871) in describing the supernumerary tendon; neither source refers to this structure as an extensor pollicis et indicis communis [13, 12]. Wood’s initial name for the structure was the “extensor primi internodii pollicis et indicis” [2]. Extensor primi internodii pollicis is the muscle we now call the extensor pollicis brevis, which was absent in the case Wood first found; thus, he considered the anomalous tendon to be taking its place. Wood at first considered the extensor primi internodii pollicis et indicis to be distinct from the extensor pollicis et indicis “by [the latter] joining the tendon of the extensor secundi internodii pollicis,” now called the EPL [12]. By 1868, however, Wood determined that these two newly described muscles were functionally the same, despite the difference in insertions along the thumb, and settled on the name extensor pollicis et indicis for both types [2]. Chiu’s other source, Macalister, agrees with this assessment and identifies a range of insertion points for the extensor pollicis et indicis; the defining factors are that the muscle originates between the EIM and EPL and continues distally until the tendon splits into two components that insert at some point along the index finger and thumb [5]. It is unclear where the “communis” in Chiu’s figure caption came from, given that neither Wood nor Macalister uses the term.

Several sources identify the EPIC as a typical muscle in other mammals, including the dog, fox, wolf, jackal, panther, and dingo, in the *absence* of an EIM or EPL [8]. In contrast, the EPIA is a human accessory muscle alongside the EIM and EPL [3]. If we were to use the name chosen by Wood, the first author to identify this structure, our body donor would have bilateral extensor pollicis et indicis muscles. However, we believe the distinction that these are accessory muscles is important; thus, we refer to them as extensor pollicis et indicis accessorius muscles and propose a corresponding amendment to the classification system described by Türker et al. Additionally, given that these accessory muscles can originate from the EPL, EIM, or the ulna itself, we suggest redefining the classification system as variants of the extensor muscles of the thumb and index finger.

The classification systems proposed by Türker et al. and Suwannakhan et al. differ fundamentally in scope, structure, and intent [9]. Türker et al. present a focused, formalized classification specifically addressing anomalies of the extensor pollicis longus (EPL), with the goal of standardizing terminology and improving clinical communication, particularly for surgical and radiologic applications. Their system organizes EPL variations into clearly defined morphological categories based on tendon presence, duplication, and aberrant course or insertion. In contrast, Suwannakhan et al. do not propose a unified classification scheme but instead provide a comprehensive descriptive framework cataloging variations across the dorsal extensor compartment of the hand. Their approach is muscle-specific and prevalence-based, detailing variations in the extensor digitorum communis, extensor indicis msucle, extensor digiti minimi, and several variant muscles, including those that secondarily involve the thumb such as the extensor pollicis et indicis. Consequently, while Türker’s system offers a concise and clinically oriented method for categorizing EPL anomalies, Suwannakhan’s work contextualizes EPL-related variants within the broader spectrum of extensor tendon anatomy, emphasizing anatomical diversity rather than classification standardization.

### Limitations of the study

The findings of the case are based on gross body donor dissection and examination, without access to clinical history, physical examination, or imaging studies such as ultrasound or CT, which could have provided insights into functional implications. Portions of the dorsal extensor retinacula were sectioned prior to identifying the accessory tendon(s). As a result, we could not definitively confirm the dorsal compartmental course of the accessory tendon(s) relative to the intact retinaculum. Although the posterior interosseous artery (PIA) typically supplies the deep extensor musculature, we were unable to document the vascular pedicle to the accessory muscle in this case. The muscular branch of PIA to the accessory muscle had been partially disrupted, and available photographs did not permit retrospective confirmation which represents another limitation of the case.

Additionally, no data were available on vascular supply, nerve conduction, genetic predisposition, or biomechanical factors that might influence the relevance of this variation in living individuals. Finally, postmortem changes and preservation techniques can alter tissue size, texture, and spatial relationships, introducing potential distortion in anatomical observations**.**

## Conclusions

Accessory muscles of the hand are relatively common, but they are often found to be unilateral. Additionally, this case presented a valuable opportunity to disambiguate the terminology around accessory extensor muscles of the hand. We believe the most accurate name for the bilateral muscle we identified is the extensor pollicis et indicis accessorius rather than the extensor pollicis et indicis communis.

Given that these muscles were found on a body donor, it is impossible to know if they held any functional significance for the patient. Patients are often unaware of accessory muscles unless they are discovered incidentally or cause pain. We hope that future research can elucidate potential clinical implications of the extensor pollicis et indicis accessorius, as well as further standardize naming conventions.

## Supplementary Information

Below is the link to the electronic supplementary material.

Supplementary Material 1

## Data Availability

No datasets were generated or analysed during the current study.
