# Correction: Bilateral extensor pollicis et indicis accessorius: clinical and anatomical perspectives

**DOI:** 10.1007/s00276-026-03905-y

**Published:** 2026-05-28

**Authors:** Oluwabusayo A. Oni, Wren Adams, Preethi Prem, Ramya M. Ramakrishnan, Antara Sira, Emily A. Vachon, Mark T. Shima, Wessam Ibrahim

**Affiliations:** Carle Illinois College of Medicine, University of Illinois Urbana-Champaign, 506 South Mathews Ave., Urbana, IL 61801 USA

**Correction to: Surgical and Radiologic Anatomy (2026) 48:115** 10.1007/s00276-026-03858-2

In this article the wrong figure caption appeared as Fig. 5; the figure caption should have appeared as shown below.

The corrected figure and caption:
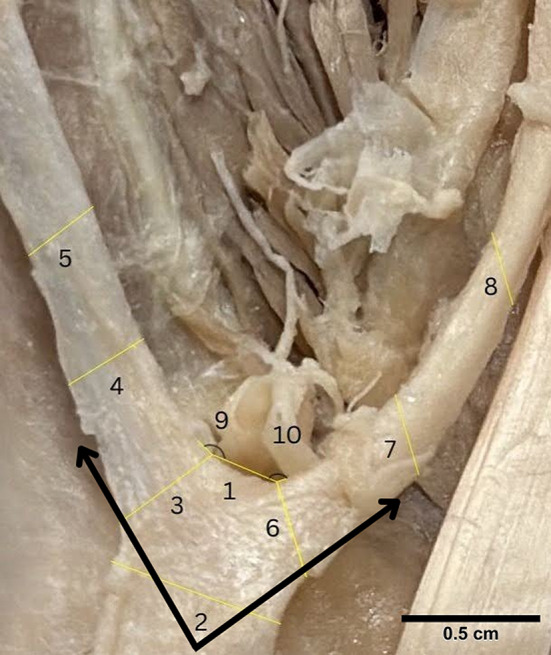


**Fig. 5** Measurements of bilateral extensor pollicis et indicis accessorius on the left hand. Numbers correspond to measurements (supplemental) White arrows point towards tendon insertion sites.

The uncorrected figure and caption:
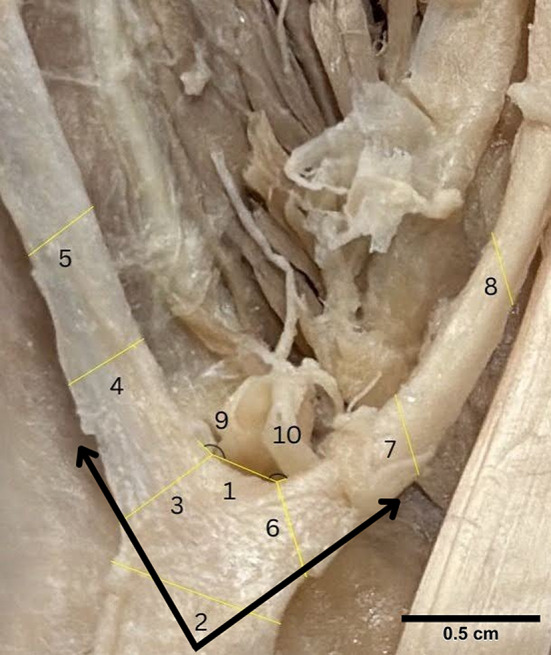


Fig. 5 White-

The original article has been corrected.

