# Supplementary material for: Bilateral extensor pollicis et indicis accessorius: clinical and anatomical perspectives
Source: Surg Radiol Anat. 2026 Apr 7;48(1):115. doi: 10.1007/s00276-026-03858-2 (PMC13056787; doi:10.1007/s00276-026-03858-2)
Supplement: Supplementary file 1 — Supplementary Material 1 [file 276_2026_3858_MOESM1_ESM.docx]

**Measurements of the Bilateral Extensor Pollicis et Indicis Accessorius on the Left and Right Hands:**

The bilateral extensor pollicis et indicis accessorius was measured using imageJ. The measurements are standardized for both extensor pollicis et indicis accessorius by having the measurements the same distance away from each other and having them be parallel to each other. A paired t-test comparing the bilateral extensor pollicis et indicis accessorius shows the measurements are not significantly different (p = 0.7466).

| \| Label # LH \| Length (cm) \| Angle (deg) \| \| --- \| --- \| --- \| \| 1 \| 0.259 \|  \| \| 2 \| 0.67 \|  \| \| 3 \| 0.424 \|  \| \| 4 \| 0.325 \|  \| \| 5 \| 0.297 \|  \| \| 6 \| 0.369 \|  \| \| 7 \| 0.305 \|  \| \| 8 \| 0.274 \|  \| \| 9 \|  \| 148.898 \| \| 10 \|  \| 141.018 \|   **Table 1** Measurements of bilateral extensor pollicis et indicis accessorius on left hand. Label number corresponds to labels of image 3 | \| Label # RH \| Length (cm) \| Angle (deg) \| \| --- \| --- \| --- \| \| 1 \| 0.255 \|  \| \| 2 \| 0.698 \|  \| \| 3 \| 0.451 \|  \| \| 4 \| 0.284 \|  \| \| 5 \| 0.231 \|  \| \| 6 \| 0.238 \|  \| \| 7 \| 0.332 \|  \| \| 8 \| 0.238 \|  \| \| 9 \|  \| 138.105 \| \| 10 \|  \| 138.517 \|   **Table 2** Measurements of bilateral extensor pollicis et indicis accessorius on right hand. Label number corresponds to labels of image 4 |
| --- | --- | --- | --- | --- | --- | --- | --- | --- | --- | --- | --- | --- | --- | --- | --- | --- | --- | --- | --- | --- | --- | --- | --- | --- | --- | --- | --- | --- | --- | --- | --- | --- | --- | --- | --- | --- | --- | --- | --- | --- | --- | --- | --- | --- | --- | --- | --- | --- | --- | --- | --- | --- | --- | --- | --- | --- | --- | --- | --- | --- | --- | --- | --- | --- | --- | --- | --- |

| 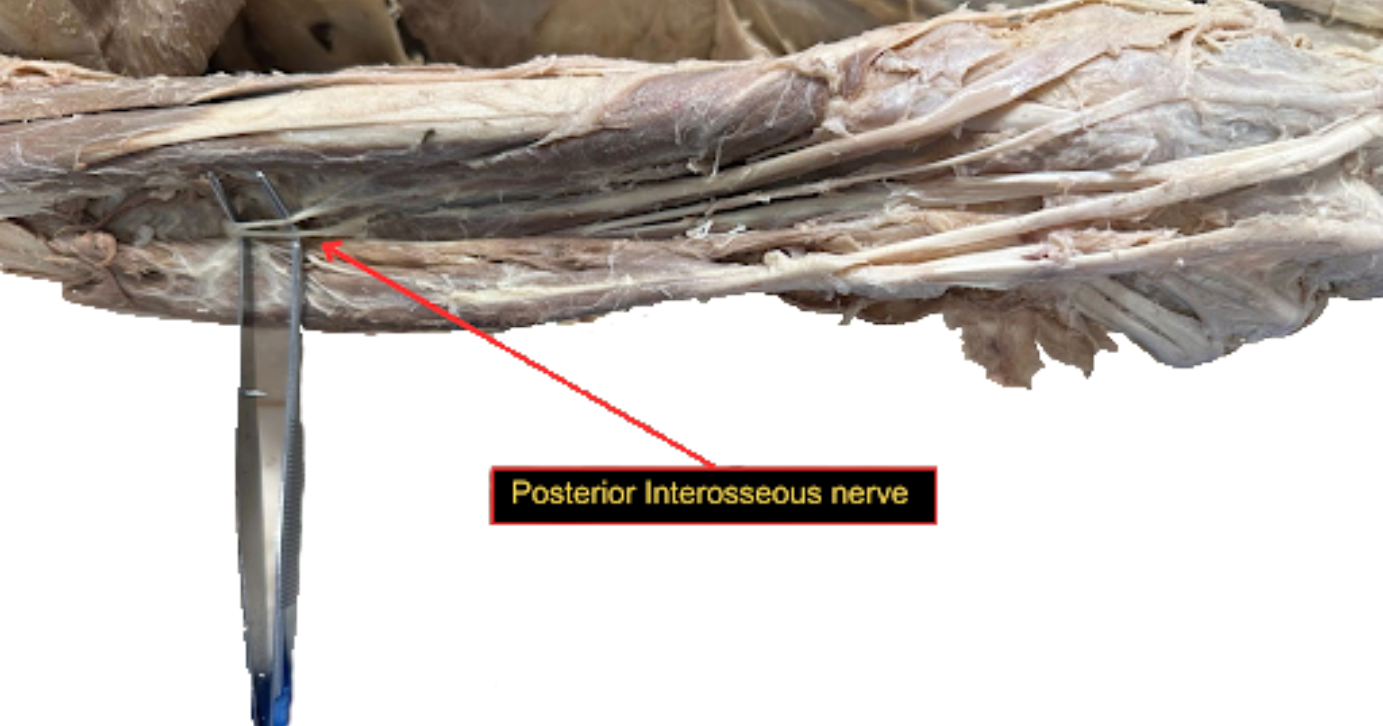 |
| --- |
| **Fig 7** Innervation of right extensor pollicis et indicis accessorius |

**Table 3 Extensor Variants Cases**

| **Author** | **Year** | **Population** | **Hands** | **Method** | **Variants Reported** | **Terminology in Source** | **Notes** |
| --- | --- | --- | --- | --- | --- | --- | --- |
| Abdel-Hamid et al. | 2013 | Egyptian adults | 95 | Cadaveric dissection | EIP, EMP, EIMC, EPI absent | EIP; EMP; EIMC; EPI | 100% EIP present; EMP/EIMC/EPI not observed |
| Agarwal & Tirthani | 2011 | Indian adults | 120 | Cadaveric dissection | EIP single/double | EIP | EIP present 97.5% |
| Bolwar | 2011 | Indian adults | 101 | Cadaveric dissection | EIP single/double | EIP |  |
| Carlos et al. | 2011 | American adults | 94 | Cadaveric dissection | EMP | EMP | EMP 7.4% |
| Cauldwell et al. | 1943 | British adults | 263 | Cadaveric dissection | EIP single/double; EIMC; EPI | EIP; EIMC; EPI |  |
| Celik et al. | 2008 | Turkish adults | 54 | Cadaveric dissection | EIP single/double; EMP; EIMC | EIP; EMP; EIMC |  |
| Dass et al. | 2011 | South Indian adults | 100 | Cadaveric dissection | EIP single/double; EMP | EIP; EMP |  |
| El-Badawi et al. | 1995 | Saudi adults | 181 | Cadaveric dissection | EIP slips incl. triple; EMP; EIMC | EIP; EMP; EIMC |  |
| Godwin & Ellis | 1992 | British adults | 50 | Cadaveric dissection | EIP; EPI | EIP; EPI |  |
| Gonzalez et al. | 1996 | American adults | 72 | Cadaveric dissection | EIP; EIMC; EPI | EIP; EIMC; EPI |  |
| Gruber | 1882 | Russian adults | 408 | Cadaveric dissection | EPI | EPI |  |
| Hirai et al. | 2001 | Japanese adults | 548 | Cadaveric dissection | EIP single/double | EIP |  |
| Komiyama et al. | 1999 | Japanese adults | 164 | Cadaveric dissection | EIP; EMP; EPI | EIP; EMP; EPI |  |
| Klena et al. | 2012 | American adults | 44 | Cadaveric dissection | EMP; EIMC | EMP; EIMC | Long finger anomalies |
| Le Double | 1897 | French adults | 164 | Cadaveric dissection | EMP | EMP | Classic monograph |
| Mestdagh et al. | 1985 | French adults | 150 | Cadaveric dissection | EIP; EMP | EIP; EMP |  |
| Mori | 1964 | Japanese adults | 205 | Cadaveric dissection | EIP single/double | EIP |  |
| Ogura et al. | 1987 | Japanese adults | 559 | Cadaveric dissection | EIP presence | EIP |  |
| Perkins & Hast | 1993 | American adults | 80 | Cadaveric dissection | EIP single/double | EIP |  |
| Ranade et al. | 2008 | Indian adults | 72 | Cadaveric dissection | EIP; EIMC | EIP; EIMC |  |
| Schenck | 1964 | American adults | 57 | Cadaveric dissection | EIMC | EIMC |  |
| von Schroeder & Botte | 1991 | American adults | 58 | Cadaveric dissection | EMP; EIMC | EMP; EIMC |  |
| von Schroeder & Botte | 1995 | American adults | 43 | Cadaveric dissection | EIP single/double/triple; EMP; EIMC | EIP; EMP; EIMC |  |
| Wagenseil | 1937 | Chinese adults | 131 | Cadaveric dissection | EPI | EPI |  |
| Wood | 1868 | British adults | 204 | Cadaveric dissection | EMP; EIMC; EPI | EMP; EIMC; EPI | Variations in human myology |
| Yalcin et al. | 2006 | Turkish adults | 62 | Cadaveric dissection | EIMC | EIMC |  |
| Yoshida | 1990 | Japanese adults | 832 | Cadaveric dissection | EIP single/double; EPI | EIP; EPI |  |
| Yoshida | 1995 | Japanese adults | 952 | Cadaveric dissection | EMP; EIMC; EPI | EMP; EIMC; EPI |  |
| Zilber & Oberlin | 2004 | French adults | 50 | Cadaveric dissection | EIP single/double; EIMC | EIP; EIMC |  |
